# XBP-1 Remodels Lipid Metabolism to Extend Longevity

**DOI:** 10.1016/j.celrep.2019.06.057

**Published:** 2019-07-16

**Authors:** Soudabeh Imanikia, Ming Sheng, Cecilia Castro, Julian L. Griffin, Rebecca C. Taylor

**Affiliations:** 1 MRC Laboratory of Molecular Biology, Francis Crick Avenue, Cambridge, UK; 2 Department of Biochemistry and the Cambridge Systems Biology Centre, University of Cambridge, 80 Tennis Court Road, Cambridge, UK

**Keywords:** *C. elegans*, aging, proteostasis, lipids, monounsaturated, metabolism, neurons, signaling

## Abstract

The endoplasmic reticulum unfolded protein response (UPR^ER^) is a cellular stress response that maintains homeostasis within the secretory pathway, regulates glucose and lipid metabolism, and influences longevity. To ask whether this role in lifespan determination depends upon metabolic intermediaries, we metabotyped *C. elegans* expressing the active form of the UPR^ER^ transcription factor XBP-1, XBP-1s, and found many metabolic changes. These included reduced levels of triglycerides and increased levels of oleic acid (OA), a monounsaturated fatty acid associated with lifespan extension in *C. elegans*. Here, we show that constitutive XBP-1s expression increases the activity of lysosomal lipases and upregulates transcription of the Δ9 desaturase FAT-6, which is required for the full lifespan extension induced by XBP-1s. Dietary OA supplementation increases the lifespan of wild-type, but not *xbp-1s*-expressing animals and enhances proteostasis. These results suggest that modulation of lipid metabolism by XBP-1s contributes to its downstream effects on protein homeostasis and longevity.

## Highlights

• *Xbp-1s* expression decreases total fat but increases oleic acid (OA) levels
• OA supplementation increases lifespan in wild-type, but not *xbp-1-s*-expressing, worms
• Increased lysosomal lipase and Δ9 desaturase activity may underlie these changes
• OA supplementation is sufficient to protect animals against proteotoxicity

## Graphical Abstract

**Figure:**
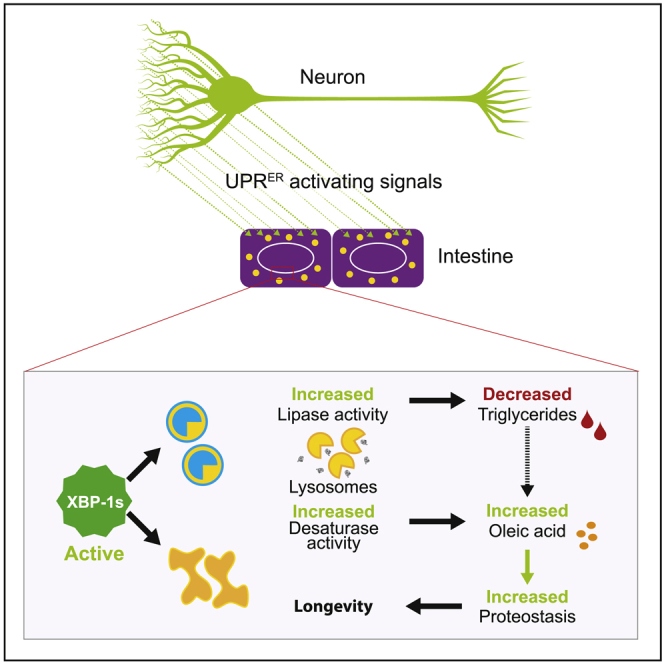

## Teaser

Imanikia et al. find that expressing the transcription factor *xbp-1s* in *C. elegans* remodels lipid metabolism, decreasing triglyceride and increasing oleic acid (OA) levels. Increased OA levels may involve the activity of lysosomal lipases and a Δ9 desaturase, and are sufficient to increase lifespan and protect animals from proteotoxicity.

## Introduction

Lifespan is not an immutable property of organisms but can be extended or reduced based on genetic or environmental factors (Finkel, 2015). One means by which lifespan can be modulated is through signals sent from neurons, which are released upon the detection of changing environmental conditions. These signals include those that activate cellular stress responses in peripheral tissues, which increase an organism’s stress resistance and protein homeostasis (proteostasis) and can also extend longevity (Taylor et al., 2014).

One of these stress responses, the endoplasmic reticulum unfolded protein response (UPR^ER^), can be activated in the intestine of *C. elegans* by signals from neurons released upon the expression of the spliced and active form of the XBP-1 transcription factor, XBP-1s, in the nervous system (Taylor and Dillin, 2013). The resulting activation of XBP-1s in the intestine leads to increased resistance to ER stress as well as extended lifespan. These cell non-autonomous properties may be conserved across species, as mice expressing Xbp1s in the pro-opiomelanocortin (POMC) neurons of the hypothalamus upregulate UPR^ER^ targets in the liver, leading to improved metabolic status as measured by glucose tolerance (Williams et al., 2014). In addition, sensing of food by POMC neurons triggers both splicing of Xbp1 and the upregulation in the liver of genes involved in lipid metabolism (Brandt et al., 2018).

A transcriptional regulator of key genes in lipid synthesis, XBP-1s has been shown to regulate glucose and lipid metabolism in mammals (Piperi et al., 2016, Lee et al., 2008). Less is known, however, about possible metabolic and lipid changes induced by this transcription factor in *C. elegans*. It is therefore not clear whether effects on metabolism might contribute to the slowed aging of worms expressing XBP-1s—a plausible scenario, as changes in metabolic state accompany many interventions that prolong life (Finkel, 2015).

We therefore decided to look at the metabolic profiles of animals expressing *xbp-1s* in either the nervous system or the intestine to explore whether this transcription factor might change metabolism to promote longevity. Using nuclear magnetic resonance (NMR) spectroscopy and gas chromatography-mass spectrometry (GC-MS) to explore levels of individual metabolites, we reveal broad metabolic changes in animals expressing *xbp-1s* in these tissues, including lower levels of total fat and significantly increased levels of a specific monounsaturated fatty acid (MUFA), oleic acid (OA), which has previously been associated with longevity in *C. elegans* (Goudeau et al., 2011, Han et al., 2017). Dietary supplementation with OA increases the lifespan of wild-type and *xbp-1* mutant worms, but not those expressing neuronal or intestinal *xbp-1s*, in which OA levels are already increased. *Xbp-1s* expression leads to increased transcription of the intestinal Δ9 desaturase *fat-6*, responsible for the conversion of stearic acid to OA, and in animals carrying mutations in fatty acid desaturases, neuronal *xbp-1s* has a reduced ability to extend longevity. In addition, lysosomal lipase activity is increased in neuronal *xbp-1s*-expressing nematodes, suggesting that this may be another mechanism through which *xbp-1s* mediates lipid remodeling. Dietary supplementation with OA bypassed the requirement for lysosomes in the improved proteostasis of these animals, suggesting that increases in OA might be a key mechanism by which *xbp-1s*-induced lysosomal activation improves proteostasis. Indeed, OA supplementation is sufficient to protect against proteotoxicity in multiple protein misfolding models, leading to increased misfolded protein clearance and reduced levels of oxidative protein damage, suggesting a mechanism by which proteostasis is improved by this MUFA. We have therefore identified an ability of the UPR^ER^ to cell non-autonomously modulate lipid metabolism, increasing OA levels and enhancing organismal proteostasis and lifespan.

## Results

**Figure 1:**
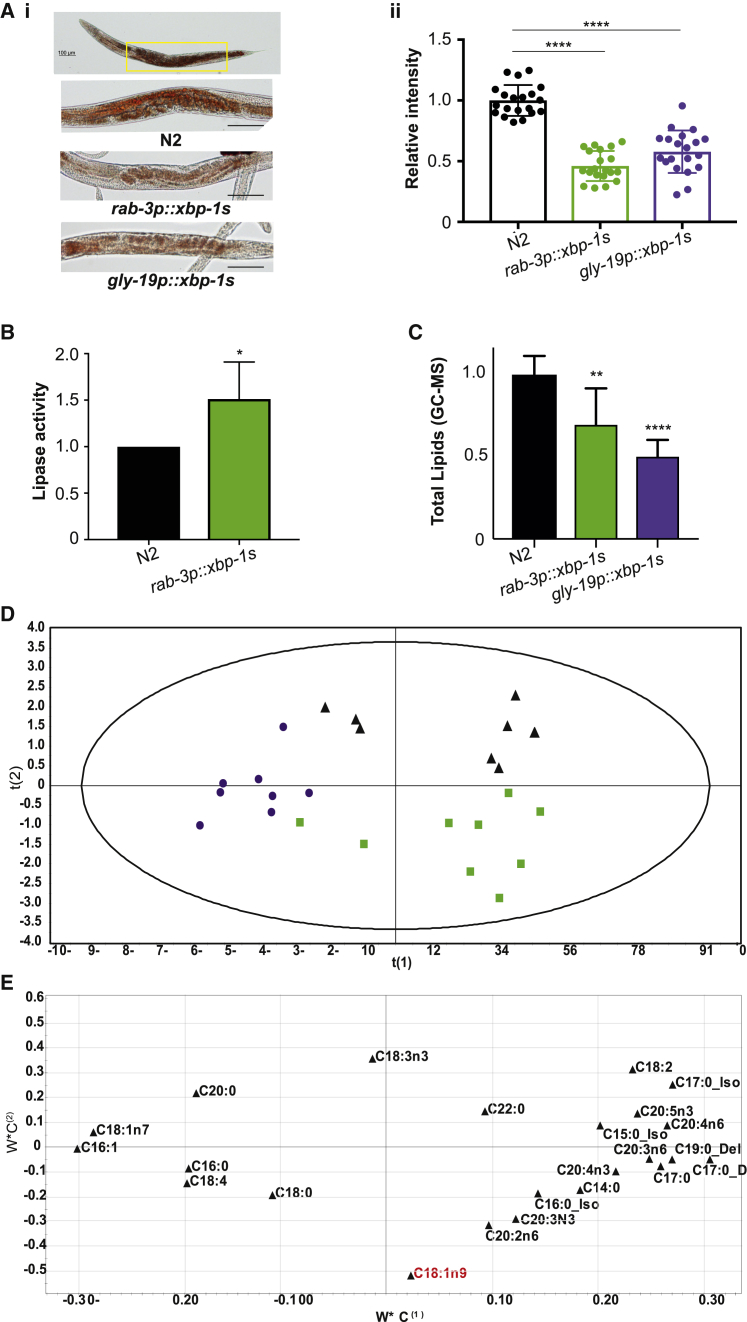
Expression of *xbp-1s* in Neurons or the Intestine Modulates Lipid Metabolism in *C. elegans* (A) (i) Oil red O (ORO) staining of total lipid droplets. Imaging was conducted in the anterior section of the intestine. Images of N2, *rab-3p::xbp-1s*, and *gly-19p::xbp-1s* were acquired at ×10 magnification in fixed animals at day 1 of adulthood. (ii) Quantification of ORO intensity from three independent experiments was carried out using ImageJ. Plots represent mean relative intensity of ORO from 18–21 animals ± SD. Statistical significance was calculated between N2 and *rab-3p::xbp-1s* or *gly-19p::xbp-1s* using one-way ANOVA with Dunnett’s multiple comparison test; ^∗∗∗∗^p < 0.0001. (B) Lipase activity in *rab-3p::xbp-1s* relative to N2 animals. Lipase activity at day 1 of adulthood was measured using a colorimetric assay in worm lysates. Bar graphs show fold change in activity relative to N2 and represent the mean ± SEM of four independent replicates. Significance was calculated by Student’s t test; ^∗^p < 0.05. (C) Total lipid levels in N2, *rab-3p::xbp-1s*, and *gly-19p::xbp-1s* animals, measured by GC-MS and normalized to N2. Data represent the mean ± SD of seven independent biological replicates. Statistical significance was calculated using one-way ANOVA with Bonferroni’s post hoc test; ^∗∗^p < 0.01 and ^∗∗∗∗^p < 0.0001. (D) Total fatty acid content plotted by partial least-squares discriminant analysis (PLS-DA) score of GC-MS data (ANOVA p value for the model 5.12E-06). Eight independent biological replicates of N2 (black triangles), *rab-3p::xbp-1s* (green squares), and *gly-19p::xbp-1s* (purple circles) are shown. (E) Loading plot of PLS-DA data showing individual fatty acids. Oleic acid (OA) (18:1n9) is highlighted in red. See also Figure S1 and Table S1.

Effects on metabolism underpin increased lifespan in many longevity-promoting paradigms. To determine whether this might be the case in long-lived *C. elegans* expressing the UPR^ER^ transcription factor *xbp-1s*, we started by staining lipids with oil red O (ORO) in animals expressing *xbp-1s* in their neurons (*rab-3p::xbp-1s*) or intestine (*gly-19p::xbp-1s*) to determine whether we could observe overall changes in levels of stored fats. We found that lipid levels were significantly reduced in the intestine of both strains, suggesting that UPR^ER^ activation modulates intestinal lipid storage both cell-autonomously and cell non-autonomously (Figure 1A). Consistent with an increase in triglyceride breakdown, we observed elevated activity of acidic lipases in *rab-3p::xbp-1s* animals, suggesting that increased lipase activity could lead to these reductions in stored fat (Figure 1B).

Lower levels of total fat were confirmed using GC-MS, revealing that both neuronal and intestinal *xbp-1s*-expressing animals contain less total lipid (Figure 1C). In addition, GC-MS analysis of individual lipid species revealed that *xbp-1s*-expressing animals have lipid profiles significantly different from wild-type (Figures 1D and 1E; Table S1). In parallel, we also explored levels of metabolites using ^1^H NMR spectroscopy, to obtain information on a broad range of molecules including amino acids, organic acids, and sugars. We found that, again, each strain differed significantly from N2 worms (Figures S1A and S1B). Some metabolic differences were shared between both genotypes, while others were unique to one of the two *xbp-1s*-expressing strains, suggesting that activation of the UPR^ER^ via neuronal signaling induces a set of metabolic changes that overlaps with but is distinct from that induced by direct intestinal *xbp-1s* expression. For example, both strains contained significantly increased levels of glutathione, but only intestinal *xbp-1s* increased succinate levels, while only neuronal *xbp-1s* altered levels of several amino acids (Figure S1B; Table S1).

We also found that neuronal, but not intestinal, *xbp-1s*-expressing animals showed an increase in the ratio of mono- to polyunsaturated fatty acids (MUFAs:PUFAs) (Figure S2A). MUFAs are particularly interesting in the context of longevity, as these lipid species have been previously implicated in lifespan extension (Han et al., 2017). To ask whether *xbp-1s*-expressing animals contain specific MUFAs, or other lipids associated with increased lifespan, we compared the fatty acid profiles of both *xbp-1s-*expressing strains with that of *daf-2(e1370)*. In these animals, insulin and insulin growth factor 1 (IGF-1)-like signaling (IIS) is reduced due to a mutation in the IIS receptor DAF-2, leading to extreme longevity and an altered lipid profile (Castro et al., 2013). Only two fatty acids were increased in all three strains: the saturated fatty acid (SFA) myristic acid and the MUFA OA (Figure S2B). The shared change in OA levels was striking, as this fatty acid has been previously connected to longevity in *C. elegans* (Gillingham et al., 2011, Goudeau et al., 2011, Han et al., 2017, Taylor and Dillin, 2013). We therefore wondered whether increased levels of OA might contribute to extended lifespan in these animals.

**Figure 2:**
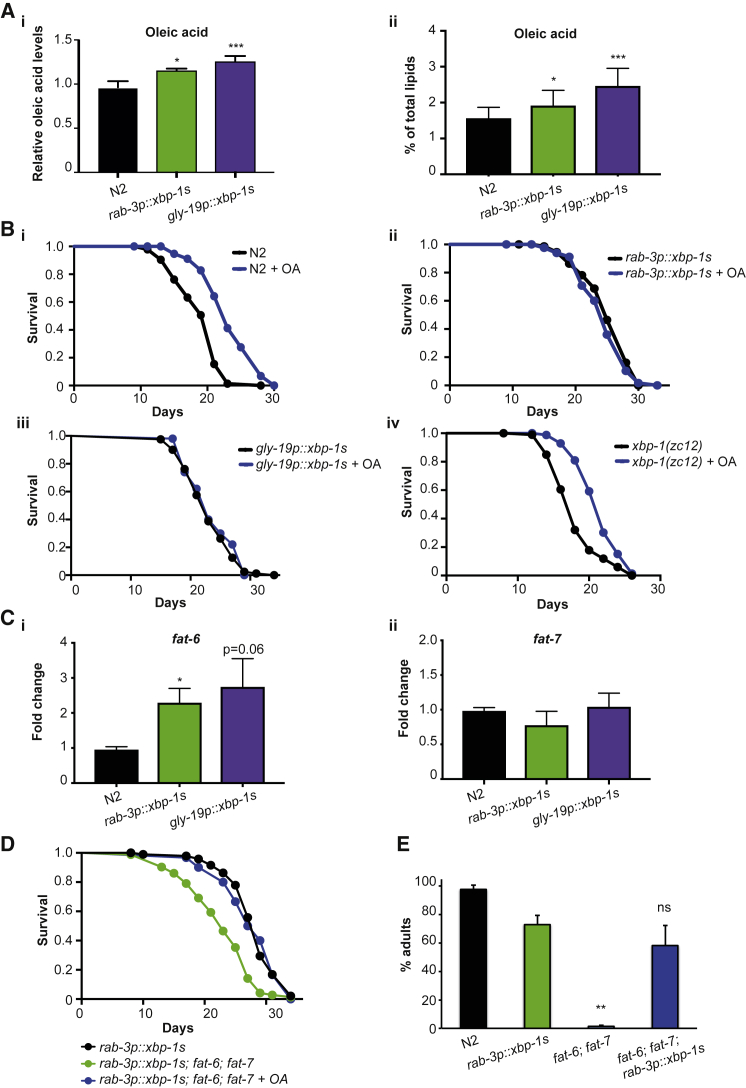
Increased OA Levels in *xbp-1s*-Expressing Animals May Play a Role in Extending Longevity (A) (i) Levels of OA in *rab-3p::xbp-1s* and *gly-19p::xbp-1s* animals measured by GC-MS and normalized to N2. Data represent the mean ± SD of seven independent biological replicates. Statistical significance was calculated using one-way ANOVA with Bonferroni’s post hoc test; ^∗^p < 0.05 and ^∗∗∗^p < 0.001. (ii) Levels of OA in *rab-3p::xbp-1s* and *gly-19p::xbp-1s* animals measured by GC-MS and expressed as percentage of total lipids. Data represent the mean ± SD of seven independent biological replicates. Statistical significance was calculated using one-way ANOVA with Bonferroni’s post hoc test; ^∗^p < 0.05 and ^∗∗∗^p < 0.001. (B) Lifespan analyses in the presence or absence of 2 mM OA. Graphs are plotted as Kaplan-Meier survival curves. p values were calculated by Mantel-Cox log-rank test. (i) N2 without OA (black), median lifespan 21 days; N2 with OA (blue), median lifespan 23 days; p < 0.0001. (ii) *rab-3p::xbp-1s* without OA (black), median lifespan 25 days; *rab-3p::xbp-1s* with OA (blue), median lifespan 25 days; p = 0.2947. (iii) *gly-19p::xbp-1s* without OA (black), median lifespan 23 days; *gly-19p::xbp-1s* with OA (blue), median lifespan 23 days; p = 0.4600. (iv) *xbp-1(zc12)* without OA (black), median lifespan 18 days; *xbp-1(zc12)* with OA (blue), median lifespan 22 days; p < 0.0001. (C) mRNA levels of (i) *fat-6* and (ii) *fat-7* measured by qRT-PCR. Transcript levels at day 1 of adulthood were assessed in *rab-3p::xbp-1s* and *gly-19p::xbp-1s* animals relative to N2. Bar graphs represent the mean ± SEM from three biological replicates. Significance was assessed by one-way ANOVA with Dunnett’s multiple comparisons test; ^∗^p < 0.05. (D) Lifespan analysis of *rab-3p::xbp1s* and *rab-3p::xbp1s*; *fat-6; fat-7* with and without 2 mM OA. Graphs plotted as Kaplan-Meier survival curves.; p values were calculated by Mantel-Cox log-rank test. *rab-3p::xbp1s* (black), median lifespan 29 days; *rab-3p::xbp-1s; fat-6; fat-7* without OA (green), median lifespan 23 days; p < 0.0001; *rab-3p::xbp-1s; fat-6; fat-7* with 2mM OA (blue), median lifespan 28 days; p = 0.8152. (E) Development to adulthood of N2, *rab-3p::xbp-1s*, *fat-6; fat-7*, and *rab-3p::xbp-1s*; *fat-6; fat-7* animals. Following 1-h timed egg lays, animals were allowed to develop at 20°C on *E. coli* OP50 for 72 h, and the percentage of animals reaching reproductive adulthood was quantified. n > 100 animals per assay; results represent the mean ± SD of three independent assays. Statistical significance was calculated between *rab-3p::xbp-1s* and *fat-6; fat-7* or *rab-3p::xbp-1s; fat-6; fat-7* using a Student’s t test; ^∗∗^p < 0.01; ns, not significant. See also Figures S2 and S3 and Table S2.

OA content in *xbp-1s*–expressing animals is significantly increased both in absolute levels and as a percentage of total lipids (Figure 2A). To ask whether increased OA might be responsible for their increased longevity, we used dietary supplementation with this fatty acid. We tested a range of OA supplementation levels (1, 2, and 4 mM) and found that, as shown previously, addition of 2 mM OA to nematode growth medium (NGM) plates was sufficient to consistently and significantly increase the lifespan of wild-type worms (Figures 2Bi and S2C; Table S2; Han et al., 2017). Supplementation with OA at this concentration increased total organismal fat content, indicative of uptake, and specifically increased OA levels in N2 animals fed upon either *E. coli* OP50 or HT115 bacteria (Figure S2D–S2F). We also confirmed that supplementation with 2 mM OA further increased the OA content of *rab-3p::xbp-1s* and *gly-19p::xbp-1s* animals (Figure S2G). However, OA supplementation did not further extend lifespan in these *xbp-1s*-expressing animals; in fact, longevity was sometimes reduced, suggesting that they already contain sufficiently elevated levels of this lipid to induce maximum lifespan extension (Figure 2Bii-iii; Table S2). In contrast, longevity of *xbp-1(zc12)* mutant animals was increased substantially by OA supplementation, suggesting that OA does not act upstream of *xbp-1* in determining longevity (Figure 2Biv; Table S2).

OA is produced by the desaturation of stearic acid, a process carried out in *C. elegans* by the Δ9 desaturases FAT-6 and FAT-7 (Figure S3A) (Brock et al., 2006). We looked at levels of *fat-6* and *fat-7* gene expression in *rab-3p::xbp-1s* and *gly-19p::xbp-1s* animals to see whether changes in transcription of these genes might account for the increased levels of OA observed in these strains. Indeed, we found that expression of the intestinal Δ9 desaturase *fat-6*, but not *fat-7*, was increased in both *rab-3p::xbp-1s* and *gly-19p::xbp-1s* animals (Figure 2C). In addition, the promoter of *fat-6* contained potential XBP-1s binding sites, including a CCACG box upstream and an ACGT core and CAAT box downstream of the *fat-6* start site, suggesting that *fat-6* may be a direct target of this transcription factor (Figure S3B), similar to the transcriptional regulation of fatty acid metabolism by XBP1s in mice (Park and Paik, 2017, Lee et al., 2008). We measured the lifespan of animals expressing neuronal *xbp-1s* in combination with mutations in *fat-6* and *fat-7* (to take into account potential redundancy between these desaturases) and found that extension of longevity by neuronal *xbp-1s* is significantly reduced in these animals but can be rescued by supplementation with 2 mM OA, suggesting that the regulation of desaturation plays a role in the ability of *xbp-1s* to modulate OA levels and extend lifespan (Figure 2D and S3C). However, suppression of longevity by *fat-6; fat-7* is not complete, implying that additional mechanisms must contribute to elevated OA in *rab-3p::xbp-1s* animals, or regulate longevity in parallel to OA, downstream of *xbp-1s*.

*fat-6; fat-7* mutant worms have an extended developmental period due to their altered lipid metabolism. By counting the number of animals reaching adulthood 72 h after egg laying, we observed that this delayed development phenotype was substantially rescued by expression of *rab-3p::xbp-1s* (Figure 2E). This suggests that expression of *xbp-1s* may utilize additional mechanisms to increase OA levels and bypass developmental defects observed in OA deficiency, compensating in part for the reduced desaturation activity of *fat-6; fat-7* mutants. One possible explanation is a change in the balance of cellular fatty acids resulting from changes in lipase activity and increased breakdown of intestinal triglycerides (Figures 1A and 1B).

**Figure 3:**
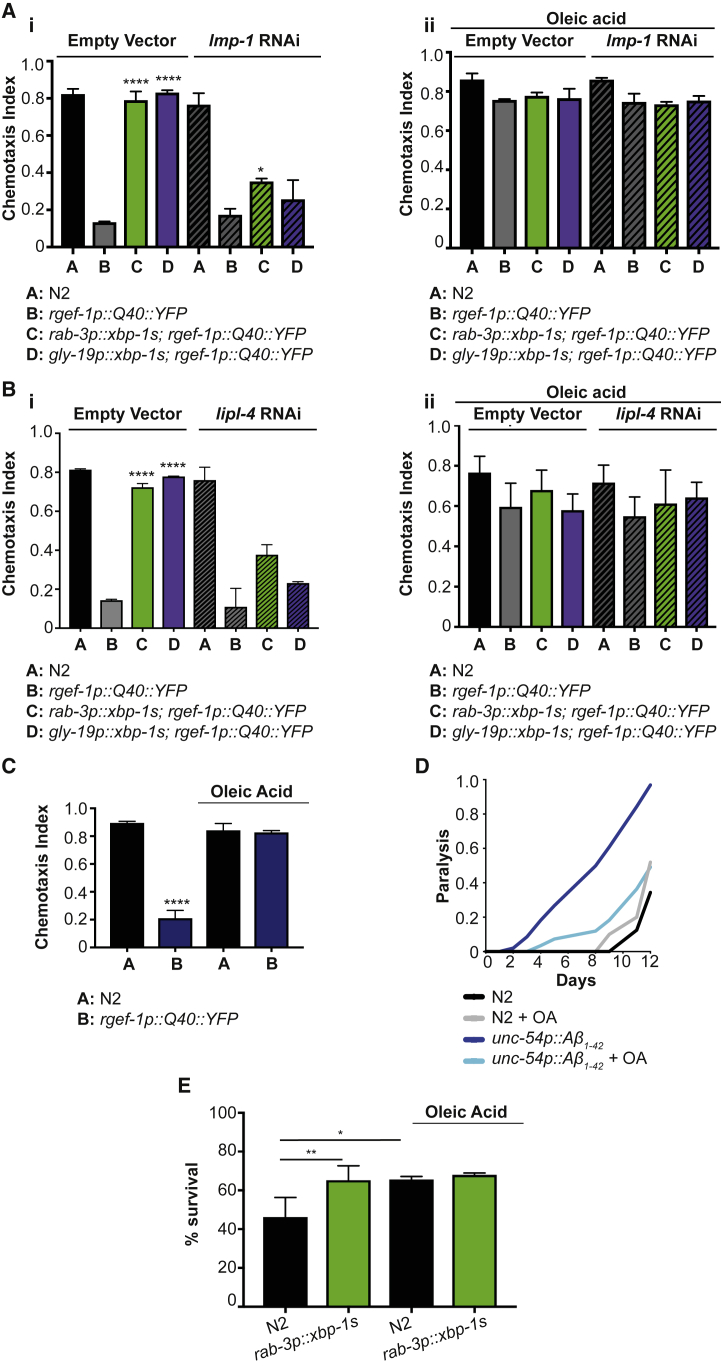
OA Supplementation Protects against Proteotoxicity and Rescues the Effect of Loss of Lysosomal Lipolysis on Proteostasis (A) (i) Chemotaxis ability in animals expressing polyQ_40_ in neurons in combination with neuronal and intestinal *xbp-1s* and *lmp-1* RNAi, in the absence of OA. Worms were grown on control (empty vector) or *lmp-1* RNAi. Bar graphs represent mean chemotaxis index ± SD; n = 60–150 animals per assay. Each assay was independently repeated three times. Significance between B and C or D in each condition was assessed by an ordinary two-way ANOVA with Tukey’s multiple comparisons test; ^∗∗∗∗^p < 0.0001. (ii) Chemotaxis ability of worms expressing neuronal polyQ_40_ in combination with neuronal and intestinal *xbp-1s* and *lmp-1* RNAi, in the presence of OA. Animals were grown on control (empty vector) or *lmp-1* RNAi, supplemented with 2 mM OA. Bar graphs represent mean chemotaxis index ± SD; n = 70–110 animals per assay. Each assay was replicated three times. Significance between B and C or D in each condition was calculated using an ordinary two-way ANOVA with Tukey’s multiple comparisons test. (B) (i) Chemotaxis ability of worms expressing neuronal polyQ_40_ in combination with neuronal and intestinal *xbp-1s* and *lipl-4* RNAi in the absence of OA. Animals were grown on control (empty vector) or *lipl-4* RNAi. Bar graphs represent mean chemotaxis index ± SD; n = 65–120 animals per assay. Each assay was replicated three times. Significance between B and C or D in each condition was calculated using an ordinary two-way ANOVA with Tukey’s multiple comparisons test; ^∗∗∗∗^p < 0.0001. (ii) Chemotaxis ability of worms expressing neuronal polyQ_40_ in combination with neuronal and intestinal *xbp-1s* and *lipl-4* RNAi in the presence of OA. Animals were grown on control (empty vector) or *lipl-4* RNAi, supplemented with 2 mM OA. Bar graphs represent mean chemotaxis index ± SD; n = 65–110 animals per assay. Each assay was replicated three times. Significance between B and C or D in each condition was calculated using an ordinary two-way ANOVA with Tukey’s multiple comparisons test. (C) Chemotaxis ability of wild-type animals versus worms expressing polyQ_40_ in neurons, with and without OA. Animals were raised on *E. coli* OP50 either in the absence or presence of 2 mM OA. Bar graphs represent mean chemotaxis index ± SD; n = 80–140 worms per assay. Each assay was replicated three times. Significance between A and B in each condition was assessed using an ordinary two-way ANOVA with Tukey’s multiple comparisons test; ^∗∗∗∗^p < 0.0001. (D) Paralysis in worms expressing Aβ_1-42_ in body wall muscle cells with and without 2 mM OA. Animals were raised on *E. coli* OP50 either in the absence or presence of 2 mM OA, and paralyzed animals were counted daily from day 1 to day 12 of adulthood. (E) Survival of N2 and *rab-3p::xbp-1s* animals, with and without 2 mM OA supplementation, following 2.5 hour heat shock at 37°C with overnight recovery. Animals were raised on *E. coli* OP50 and subjected to heat shock at day 1 of adulthood. Bar graphs represent mean survival ± SD. Significance was assessed by one-way ANOVA with Tukey’s multiple comparisons test; ^∗^p < 0.05 and ^∗∗^p < 0.01. See also Figure S4.

We have shown that lysosome activity is increased in animals expressing neuronal *xbp-1s* and that lysosome function is required for *xbp-1s*-mediated extension of lifespan and protection against proteotoxicity (Imanikia et al., 2019). To explore the possibility that lipid remodeling through enhanced activity of lysosomal lipases plays a role downstream of *xbp-1s*, we asked whether supplementation with OA could rescue loss of lysosome function in proteotoxicity assays. We have previously established that expression of *xbp-1s* in neurons renders animals more resistant to the toxicity caused by exogenous expression of disease-associated proteins, including proteotoxic polyglutamine expansions associated with neurodegenerative disease, a resistance that is dependent upon lysosome function. We therefore decided to determine the effect of OA supplementation on loss of neuronal cell function in animals expressing polyglutamine expansions in the nervous system by assaying chemotaxis, the ability to move toward an attractive volatile odorant (Gidalevitz et al., 2006, Brignull et al., 2006). Loss of chemotaxis in animals expressing pan-neuronal Q40::YFP can be rescued by expression of neuronal or intestinal *xbp-1s*, dependent upon lysosome function; knockdown of the *C. elegans* homolog of the lysosomal gene LAMP1, *lmp-1*, abolishes protection against proteotoxicity in *xbp-1s*-expressing animals. Supplementation with either 1 or 2 mM dietary OA eliminates this requirement for lysosome function (Figures 3A and S4A). In addition, we found that activity of the lysosomal lipase LIPL-4, previously implicated in regulation of longevity by lysosomes (Folick et al., 2015), was required for the full protective effect of *xbp-1s*; again, this requirement could be bypassed by addition of OA (Figure 3B). This suggests that changes in lipid balance may be important downstream of lysosome activation in protection against proteotoxicity. However, OA was not able to compensate for the loss of chemotaxis resulting from knockdown of *xbp-1* itself, suggesting that this molecule is not sufficient to fully compensate for the collapse in proteostasis suffered in the absence of *xbp-1* and that other parallel downstream factors must be important (Figure S4B).

This analysis suggested that OA supplementation not only was able to rescue the lysosome dependency of *xbp-1s*-mediated protection from proteotoxicity but also was sufficient to protect against neuronal proteotoxic species directly. We confirmed that 2 mM OA supplementation could protect against proteotoxicity mediated by neuronal Q40::YFP (Figure 3C). To determine whether OA supplementation was also sufficient to protect against other proteotoxic species, we asked whether it had a beneficial effect on worms expressing the human Aβ_1–42_ peptide in body wall muscle cells (Cohen et al., 2006, Link, 1995). Age-related paralysis was substantially mitigated by supplementation with OA in these animals (Figure 3D). However, OA supplementation was not universally protective; in animals expressing Aβ_1–42_ in the nervous system (Wu et al., 2006), the addition of OA was unable to rescue chemotaxis (Figure S4C). This suggests that elevated OA levels can directly protect against the toxicity associated with some, but not all, proteotoxic species.

We then asked whether OA could protect animals from an acute proteotoxic stress that causes misfolding of endogenous proteins. We measured survival following heat shock (Zevian and Yanowitz, 2014) and found that OA or neuronal *xbp-1s* expression significantly protected against heat-shock-induced death; however, as with lifespan, OA could not further improve the enhanced resistance of *xbp-1-s*-expressing animals (Figure 3E). This confirms that OA can protect from proteotoxic stress in a manner that is not additive with constitutive *xbp-1s* expression.

**Figure 4:**
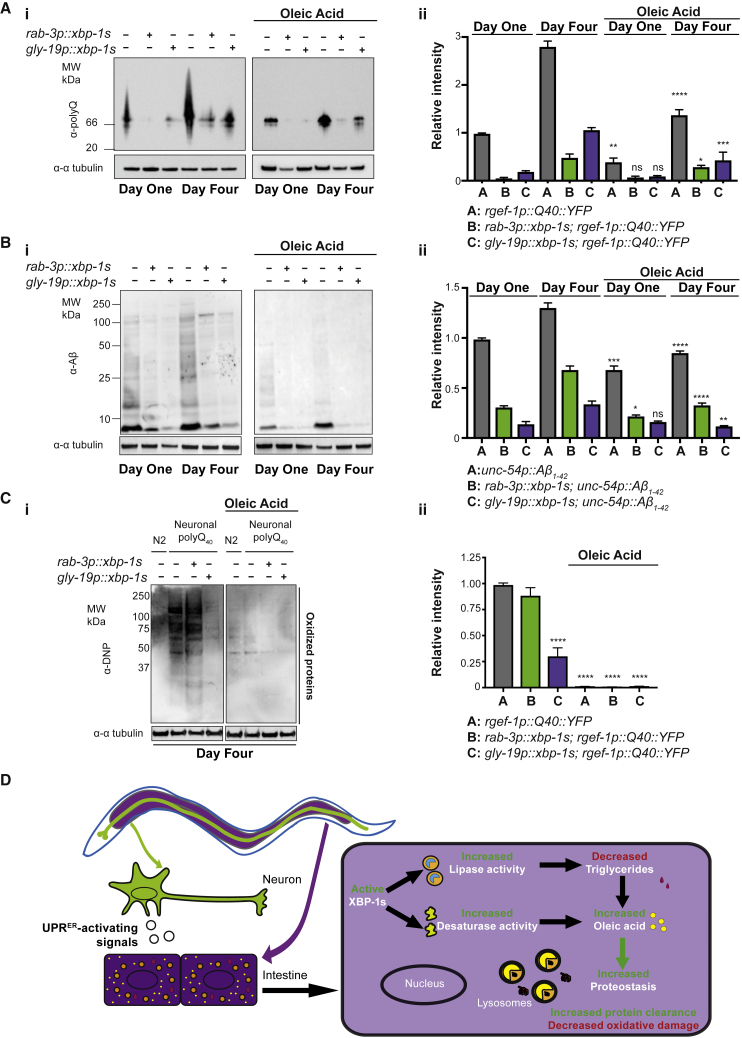
OA Reduces Levels of Proteotoxic Species and Oxidized Proteins (A) (i) Western blot analysis of neuronal polyQ_40_::YFP, expressed with and without tissue specific *xbp-1s*, at day 1 and day 4 of adulthood in the presence and absence of 2 mM OA. Lysates containing total proteins were resolved under native conditions and probed with an anti-polyQ antibody. Tubulin levels were probed with α-α tubulin as a loading control. Data represent three independent experiments. (ii) Quantification of western blots shown in (i) using ImageJ software. Bar graphs represent mean band intensity relative to day 1 *rgef-1p::Q40::YFP* ± SD. Statistical significance of intensity differences between oleic-acid-supplemented lanes versus equivalent non-supplemented lanes was assessed using two-way ANOVA with Tukey’s multiple comparisons; ^∗^p < 0.05, ^∗∗^p < 0.01, ^∗∗∗^p < 0.001, and ^∗∗∗∗^p < 0.0001. (B) (i) Western blot analysis of muscle Aβ_1–42_, expressed with and without tissue-specific *xbp-1s*, at day 1 and day 4 of adulthood in the presence and absence of 2mM OA. Lysates containing total proteins were resolved under native conditions and probed with an anti-YFP and GFP antibody. Tubulin levels were probed with α-α tubulin as a loading control. Data represent 3 independent experiments. (ii) Aβ_1–42_ was quantified from western blots shown in (i) using ImageJ software. Bar graphs represent mean band intensity relative to day 1 *unc-54p::Aβ*_*1-42*_ ± SD. Statistical significance of intensity differences between OA-supplemented lanes versus equivalent non-supplemented lanes was assessed using two-way ANOVA with Tukey’s multiple comparisons; ^∗^p < 0.05, ^∗∗^p < 0.01, ^∗∗∗^p < 0.001, and ^∗∗∗∗^p < 0.0001. (C) (i) Oxyblot analysis of worms with polyQ_40_::YFP expressed in neurons, with and without tissue-specific *xbp-1s*, at day 1 and day 4 of adulthood in the presence and absence of OA. Blots were probed with an α-dinitrophenyl (DNP) antibody, which recognizes 2,4-dinitrophenylhydrazone formed from a 2,4-dinitrophenylhydrazine (DNPH) reaction with carbonyl groups, to indicate oxidized proteins. α-α tubulin was used to verify equal loading. Data represent three independent experiments. (ii) α-DNP was quantified from Oxyblots shown in (C) using ImageJ software. Bar graphs represent mean lane intensity ± SD relative to day 1 *rgef-1p::Q40::YFP* without OA. Statistical significance was assessed using two-way ANOVA with Tukey’s multiple comparisons; ^∗∗∗∗^p < 0.0001. (D) A model for the role of OA downstream of *xbp-1s* expression. See also Figure S4.

To explore the mechanism by which OA protects against proteotoxicity, we used western blotting to determine whether levels of neuronal Q40::YFP were altered by OA supplementation. Like *xbp-1s* expression, addition of OA significantly reduced levels of Q40::YFP, suggesting that this lipid may act downstream of *xbp-1s* in mediating enhanced clearance of proteotoxic species (Figures 4A and S4D). In particular, OA inhibited the age-dependent accumulation of Q40::YFP, suggesting that it may help to preserve a more youthful proteostasis state. Similarly, OA supplementation reduced levels of Aβ_1–42_ when this peptide was expressed in muscles, mirroring its ability to protect against proteotoxicity in this model (Figure 4B). Transcript levels of polyQ::YFP and Aβ_1–42_ were not reduced upon OA supplementation, suggesting that reduced protein levels are likely to be mediated through changes in protein clearance rather than synthesis (Figure S4E). One interesting observation was that dietary OA further reduces levels of proteotoxic species in animals expressing *rab-3p::xbp-1s* or *gly-19p::xbp-1s*, without increasing lifespan in these strains, demonstrating that these phenotypes are not always fully correlated and can be uncoupled.

Finally, OA addition was able to very effectively suppress the increased levels of proteome-wide oxidative damage observed upon expression of Q40::YFP (Figure 4C). This suggests that OA may reduce oxidative damage to proteins or increase clearance of oxidatively damaged species, demonstrating that increases in OA levels can directly improve proteostasis and lead to reduced levels of toxic and damaged proteins (Figure 4D).

## Discussion

Metabolic mechanisms have been implicated in many paradigms of extended longevity. We explored the metabolic changes occurring in long-lived *C. elegans* expressing the UPR^ER^ transcription factor *xbp-1s* in the intestine or nervous system and found that expression of *xbp-1s* causes substantial changes to organismal metabolites, including reductions in stored fats and an altered lipid profile. One of these changes is an increase in levels of the MUFA OA, potentially resulting from an increase in the activity of lysosomal lipases that break down stored fats in the intestine, alongside an XBP-1s-induced increase in expression of the intestinal Δ9 desaturase FAT-6. Supplementation with OA bypasses the requirement for lysosomes and lysosomal lipases in *xbp-1s*-induced protection against proteotoxicity and, surprisingly, is sufficient to protect against the toxicity associated with neuronal polyQ expansions and other, but not all, proteotoxic species. This protection is associated with a decrease in levels of proteotoxic species and oxidative protein damage. Therefore, a pathway typically thought of as a guardian of protein homeostasis may in fact mediate its effects on proteotoxicity and lifespan at least in part by remodeling organismal lipid metabolism.

Increased levels of MUFAs have been previously associated with improved health and longevity (Gillingham et al., 2011). In *C. elegans*, dietary supplementation with MUFAs, including OA, significantly extends lifespan, and endogenous increases in levels of OA and related molecules have been connected to various longevity-associated signaling pathways, suggesting that increased OA may represent a core downstream longevity mechanism (Goudeau et al., 2011, Han et al., 2017, Folick et al., 2015). Modifiers of H3K4me3 specifically increase MUFAs, including OA, through increased expression of the desaturases *fat-5* and *fat-7*, and this increased desaturation is required for extended lifespan in H3K4me3-methyltransferase-deficient worms (Han et al., 2017). Overexpression of the lysosomal lipase LIPL-4 extends longevity; this lifespan extension depends upon increased levels of the OA-related lipid oleoylethanolamine, which increases lifespan through interaction with the nuclear hormone receptors (NHRs) NHR-80 and NHR-49 (Folick et al., 2015). In *glp-1* animals, in which signals from the germline extend longevity, *fat-6* transcript levels are upregulated by NHR-80, leading to the desaturation of stearic acid to OA (Goudeau et al., 2011). Increased OA is required for full lifespan extension of *glp-1* worms but is not sufficient to compensate for loss of *nhr-80*, suggesting that OA acts in concert with other targets of this NHR.

This has striking parallels with our model, in which production of OA may contribute to full longevity and enhanced proteostasis downstream of signals sent by *xbp-1s*-expressing neurons but cannot compensate for loss of *xbp-1*. In addition, further increasing levels of OA in animals where this molecule is already enriched does not further increase lifespan, even though misfolded proteins are cleared at higher rates. This strongly suggests that other downstream *xbp-1* targets and mechanisms are also required for longevity. It is possible that other lysosomal mechanisms, such as enhanced lysosomal proteolysis, are also critical and act in concert with or in parallel to changes in lipid metabolism downstream of lysosomes; other, yet to be identified non-lysosomal mechanisms may also be required downstream of *xbp-1s*.

We show here that supplementation with OA can directly influence how a cell handles toxic proteins. This ability of OA to improve resistance to proteotoxic species reveals the possibility of an intimate link between proteostasis and lipid metabolism in the context of UPR^ER^ activation and suggests a means by which increased levels of this fatty acid may improve health and longevity. Changes in fat metabolism have been previously linked to protein dynamics in neurodegenerative disease (Sharon et al., 2003). There is also existing evidence for a connection between lipid metabolism and proteostasis in *C. elegans*; overexpression of LIPL-4 enhances heat shock response activation and resistance to proteotoxic species, and dietary supplementation with arachidonic acid, present in elevated levels in these animals, can recapitulate these effects (Shemesh et al., 2017b). Furthermore, developmental supplementation with dihomo-γ-linolenic acid can also benefit proteostasis (Shemesh et al., 2017a). In this case, however, enhanced proteostasis does not lead to lifespan extension, adding to growing evidence that these phenotypes, while often closely correlated, can be uncoupled (van Ham et al., 2010, Volovik et al., 2014).

Our results suggest that manipulation of cellular OA content might represent a means to prevent or treat proteotoxic disease. However, the underlying mechanisms by which these effects on proteostasis are mediated are not yet clear. Our data show increased clearance of toxic species upon OA supplementation and suggest that the presence of OA reduces oxidative protein damage, consistent with other studies that have shown a role for OA in resistance to oxidative stress (Haeiwa et al., 2014). One possibility is that reduced oxidative damage renders proteins easier to clear or reduces the load on the protein clearance machinery. Other possibilities include the activation of NHRs by this fatty acid, increases in membrane fluidity that change the localization of protein aggregates or the susceptibility to damage of cell membranes, or a role for OA as an intercellular signaling molecule. These possibilities represent interesting and potentially therapeutically relevant targets for future research into mechanisms underlying aging and the maintenance of proteostasis.

## STAR★Methods

### Key Resources Table

**Table:** 

REAGENT or RESOURCE	SOURCE	IDENTIFIER
**Antibodies**		

α-Aβ (6E10)	BioLegend	6E10; Cat#803001; RRID: AB_2564653
α-polyQ (3B5H10)	Sigma Aldrich & Millipore	3B5H10; Cat#MABN821; RRID: AB_532270
α-GFP/YFP (3H9)	ChromoTek	3H9; Cat#3h9-100; RRID: AB_10773374
α-α-tubulin (T5168)	Sigma Aldrich	B512; Cat#T5168; RRID: AB_477579

**Bacterial and Virus Strains**		

*E. coli* OP50	CGC	WB OP50; RRID:WB-STRAIN:OP50
*E. coli* HT115	CGC	WB HT115; RRID:WB STRAIN:HT115
L4440 RNAi	Addgene	Cat#1654
*lmp-1* RNAi	Ahringer library, Source Bioscience	Cat#3318_Cel_RNAi_complete
*xbp-1* RNAi	Vidal ORF-RNAi library, Source Bioscience	Cat#3320_Cel_ORF_RNAi
*lipl-4* RNAi	Ahringer library, Source Bioscience	Cat#3318_Cel_RNAi_complete

**Chemicals, Peptides, and Recombinant Proteins**		

Oil Red O	Sigma Aldrich	Cat#O0625
Oleic acid	Sigma Aldrich	Cat#O1383
Oleic acid - 13C18	Sigma Aldrich	Cat#490431
Benzaldehyde	Sigma Aldrich	Cat#B1334-250ML
SYBRGreen Mastermix	Applied Biosystems	Cat#4472897

**Critical Commercial Assays**		

QuantiChrom Colorimetric Lipase Activity Assay	BioAssay Systems	Cat#DLPS-100
M-MLV Reverse Transcription Kit	Promega	Cat#M1701
OxyBlot Kit	Merck Millipore	Cat#S7150
Pierce BCA Protein Assay Kit	Thermo Scientific	Cat#23227

***C. elegans* strains**		

Wild type, Bristol	CGC [Brenner, 1974]	N2
uthIs270[*rab-3p::xbp-1s, myo-2p::tdTomato*]	CGC [Taylor and Dillin, 2013]	AGD927
*fat-6(tm331); fat-7(wa36)*	CGC [Brock et al., 2006]	BX156
dvIs50[*snb-1p::Aβ_1-42_, mtl-2p::GFP*]	CGC [Wu et al., 2006]	CL2355
dvIs2[*unc-54p::Aβ_1-42_, rol-6(su1006)*]	CGC [Link, 1995]	CL2006
rmIs110[*rgef-1p::Q40::YFP*]	[Imanikia et al., 2019]	AGD1397
*xbp-1(zc12)*	[Taylor and Dillin, 2013]	AGD1049
uthIs388[*gly-19p::xbp-1s, myo-2p::tdTomato*]	[Imanikia et al., 2019]	AGD1379
uthIs270[*rab-3p::xbp-1s*, *myo-2p::tdTomato*]; rmIs110[*rgef-1p::Q40::YFP*]	[Imanikia et al., 2019]	AGD1399
uthIs388[*gly-19p::xbp-1s*, *myo-2p::tdTomato*]; rmIs110[*rgef-1p::Q40::YFP*]	[Imanikia et al., 2019]	RCT31
uthIs270[*rab-3p::xbp-1s, myo-2p::tdTomato*]; dvIs2[*unc-54p::Aβ1-42, rol-6(su1006)*]	[Imanikia et al., 2019]	RCT1
uthIs388[*gly-19p::xbp-1s, myo-2p::tdTomato*]; dvIs2[*unc-54p::Aβ1-42, rol-6(su1006)*]	[Imanikia et al., 2019]	RCT2
uthIs270[*rab-3p::xbp-1s, myo-2p::tdTomato*]; *fat-6(tm331); fat-7(wa36)*	This paper	RCT63

**Oligonucleotides**		

See Table S3		

**Software and Algorithms**		

Chenomx NMR Suite 5.0	Chenomx, Alberta, Canada	
Xcalibur Version 2.0	Thermo Fisher	

### Lead Contact and Materials Availability

Further information and requests for resources and reagents should be directed to and will be fulfilled by the Lead Contact, Rebecca Taylor (rtaylor@mrc-lmb.cam.ac.uk).

### Experimental Model and Subject Details

*C. elegans* strains were maintained at 20°C on nematode growth medium (NGM) plates seeded with OP50 bacteria unless otherwise stated (Brenner, 1974). For feeding RNAi experiments, either L4440 empty vector or the designated RNAi bacteria was used (Kamath et al., 2000). Plates for RNAi analysis were prepared by supplementation of agar with 100 μg/mL carbenicillin (Formedium) and 1mM IPTG (Generon) after autoclaving. 24 hours prior to each assay plates were spotted with 100 μL of overnight bacterial culture.

### Method Details

#### Oil-Red-O staining

Age-matched nematodes were grown to day 1 of adulthood, then rinsed with 1x PBS, collected in 1.5 mL Eppendorf tubes, and washed twice with 1x PBS. After the final wash, supernatant was reduced to 120 μL and an equal volume of 2x MRWB (160 mM KCl, 40 mM NaCl, 14 mM Na_2_EGTA, 1 mM spermidine-HCl, 0.4 mM spermine, 30 mM Na-PIPES pH 7.4, 0.2% β-mercaptoethanol) was added along with 2% paraformaldehyde (PFA). Samples were incubated at room temperature on a rocker for 1 hour. Worms were then allowed to settle by gravity and washed with 1x PBS to remove PFA. To dehydrate the samples, 60% isopropanol was added and incubated at room temperature for 15 minutes. Oil-Red-O stock solution was prepared beforehand by dissolving the dye in isopropanol to a stock concentration of 0.5 mg/mL. Before use, Oil-Red-O was diluted with water to reach a final concentration of 60%, filtered, and 1 mL of dye added to each sample under rotation at room temperature overnight. Dye was then removed and worms washed prior to imaging. Animals were mounted on 2% agarose pads and imaged at 10x magnification using an OLYMPUS BX41 with DIC optics connected to a NIKON Digital Sight. Image quantification was performed using ImageJ.

#### Lipase assays

Worms were synchronized by bleaching and allowed to grow on NGM plates seeded with OP50 at 20°C until day 1 of adulthood. Populations were then harvested and washed in 3 × 15 mL of M9 buffer. Samples were flash frozen in liquid nitrogen prior to use and, once thawed, animals were mechanically disrupted using a Precellys (Bertin Instruments) programmed for 3x15 s pulses, with 30 s between each pulse. Samples were then centrifuged for 5 minutes at 10000 g and the protein content of the supernatant measured using a Pierce BCA protein assay kit (Thermo Scientific). Lysates were normalized according to protein content and the lipase activity of each sample measured using a QuantiChrom colorimetric lipase activity assay (BioAssay Systems). Activity was measured in 4 independent biological replicates per genotype.

#### Metabolite extraction procedure

Metabolites from whole *C. elegans*, ∼400,000 worms per sample, grown at 20°C were extracted using methanol-chloroform. 600 μL of a methanol-chloroform mix (2:1, v/v) was added to frozen nematodes. Samples were homogenized using a Polytron homogenizer and sonicated for 15 min. 200 μL each of chloroform and water were added, and the samples centrifuged to separate the aqueous from the lipid layer. The procedure was repeated twice. The aqueous layers were pooled and then dried overnight in an evacuated centrifuge, while the organic layers were pooled and dried overnight under a stream of nitrogen gas.

#### GC-MS

Lipid extracts were dissolved in 200 mL of chloroform–methanol (1:1 v/v) and half of it used for GC-MS analysis of total fatty acids. 50 μL of D-25 tridecanoic acid (200mM in chloroform) as internal standard, 650 μL of chloroform–methanol (1:1 v/v) and 250 μL BF_3_/methanol (Sigma-Aldrich) were added to the extract and vials then incubated at 80°C for 90 min. 500 μL H_2_O and 1 mL hexane were added and after vortexing two fractions were obtained. The organic layers were separated and evaporated to dryness before reconstitution in 100 mL hexane for analysis. 2 μL of the derivatized organic metabolites were injected onto a TR-fatty acid methyl ester (FAME) stationary phase column (Thermo Electron; 30 m∗0.25mm ID∗0.25mm; 70% cyanopropyl polysilphenylene–siloxane) with a split ratio of 20. The injector temperature was 230°C and the helium carrier gas flow rate was 1.2 mL min^-1^. The column temperature was 60°C for 2 min, increased by 15°C min^-1^ to 150°C, and then increased at a rate of 4°C min^-1^ to 230°C (transfer line = 240°C; ion source = 250°C, EI = 70 eV). The detector was turned on after 240 s, and full-scan spectra were collected using 3 scans per s over a range of 50–650 m/z. Peaks were assigned using the Food Industry FAME Mix (Restek 6098) and the Bacterial Acid Methyl Ester (BAME) Mix solution (Supelco 47080). GC–MS chromatograms were analyzed using Xcalibur, version 2.0 (Thermo Fisher), integrating each peak individually. Peaks were normalized to total area.

#### NMR spectroscopy

Dried aqueous extracts were rehydrated in 600 μL D_2_O containing 0.05 mM sodium-3-(tri-methylsilyl)-2,2,3,3-tetradeuteriopropionate (TSP) (Cambridge Isotope Laboratories, MA, USA) as an internal standard. Samples were analyzed using an AVANCE II+ NMR spectrometer operating at 500.13 MHz for the ^1^H frequency (Bruker, Germany) using a 5 mm TXI probe. Proton NMR spectra were collected using a solvent suppression pulse sequence based on a one-dimensional NOESY pulse sequence to saturate the residual ^1^H water signal (relaxation delay = 2 s, t_1_ increment = 3 μs, mixing time = 150 ms, solvent presaturation applied during the relaxation time and the mixing time). 196 transients were collected into 16 K data points over a spectral width of 12 ppm at 27°C. In addition, representative samples of nematodes were also examined by two-dimensional spectroscopy, including COSY (COrrelation SpectroscopY), in conjunction with previous literature and databases and the Chenomx spectral database contained in Chenomx NMR Suite 5.0 (Chenomx, Alberta, Canada) for spectral assignment. The integrals of the different metabolites were obtained using Chenomx, and normalized to total area.

#### Multivariate analysis of metabolic profiles

Each set of metabolic profiles obtained was analyzed by multivariate analysis. Datasets were imported into SIMCA-P 15.0 (Umetrics) for processing using PCA and PLS-DA (a regression extension of PCA used for supervised classification). Proton NMR data were Pareto scaled, in which each variable was centered and multiplied by 1/(*S*_*k*_)^1/2^ where *S*_*k*_ is the s.d. of the variable.

#### RNA extraction and qPCR

Total RNA was extracted from populations of 1500-3000 worms using a phenol-based extraction procedure (Tri-reagent, Sigma). RNA was purified on an RNeasy mini column (QIAGEN). Using 1 μg of RNA, cDNA was synthesized with an M-MLV reverse transcription kit (Promega). SYBRGreen quantitative RT-PCR was performed using the Corbett system and following the Rotor-Gene 6000 Series Software manual. Data from 3 biological repeats were analyzed using the comparative 2ΔΔCt method and significance assessed by one way ANOVA with Dunnett’s multiple comparisons test.

#### Oleic acid supplementation

Oleic acid (oleic acid-^13^C_18_ for experiments involving labeled OA) at a final concentration of 1 mM, 2 mM, or 4 mM was added to molten NGM agar and plates were stored in the dark at 4°C. For RNAi, carbenicillin and IPTG were also added.

#### Lifespan analysis

Lifespan analyses were performed at 20°C and were repeated at least 3 times. A minimum of 100 animals was used per condition, and worms were scored for viability every second day, from day 1 of adulthood (treating the pre-fertile day preceding adulthood as t = 0). Lifespans were performed on *E. coli* OP50, unless otherwise indicated, and animals were treated with 100 μg/mL FUDR at t = 0 and again at day 5 of adulthood. Prism 7 software was used for statistical analysis, and P values were calculated using the log-rank (Mantel–Cox) method.

#### Development assays

Worms were age-synchronized by 1 hour timed egg lay and allowed to develop at 20°C on NGM plates seeded with OP50. 72 hours after the end of the timed egg lay, the number of adult and pre-adult animals was counted, adulthood being defined by adult vulval morphology and the presence of eggs in the reproductive tract. The percentage of adult animals out of total worm number was then calculated. Each assay was repeated at least 3 times with at least 100 animals per genotype in each replicate.

#### Chemotaxis assays

Animals were synchronized by timed egg lay on 55 mm NGM plates. Chemotaxis assays were conducted at day 1 of adulthood as described (section 4.4 of Hart, 2006). Briefly, animals were raised on OP50 or RNAi bacteria to day 1 of adulthood, when they were collected and washed three times using M9 buffer, with worms settled between washes by gravity. Assay plates were prepared using a 1 μL spot of ethanol (solvent spot) and a 1 μL spot of chemo-attractant, 1:100 benzaldehyde (Sigma Aldrich) (target spot) on opposite sides. 1 μL of sodium azide (50mM) was added to solvent and target spots once dried. Worms were dispensed at the center of the plate and animals were kept in the dark for 60 min at room temperature before worms at each spot were counted. Chemotaxis indices were then calculated as (# worms at target spot - # worms at vehicle spot)/total number of worms on the plate. Indices were reported as −1 to 1, where 1 indicates that 100% of animals have arrived at the target spot.

#### Paralysis assays

Eggs were isolated by bleaching and allowed to develop on a bacterial lawn. At the L4 larval stage, individual nematodes were picked and transferred to 55mm NGM plates seeded with OP50. Paralysis was evaluated daily from day 1 to day 12 of adulthood and scored by touching the animal’s nose with a platinum wire. Worms able to move the head but not the rest of the body were scored as paralyzed (Cohen et al., 2006). Dead animals or those with other phenotypes (e.g., vivipary) were censored from the analysis. All assays were performed with at least 100 animals and repeated at least 3 times.

#### Heat stress assays

Animals were age synchronized by timed egg lay and allowed to develop on plates with or without oleic acid supplementation. At the L4 larval stage, individual worms were picked onto a fresh plate (20 worms/plate) and raised to day 1 of adulthood. Animals were exposed to 37°C for 2.5 hours in a water-bath, followed by overnight recovery at 20°C. The following day, survival rate was calculated by counting the number of live versus dead worms.

#### Western Blotting

For native extracts, nematodes were synchronized by bleaching and allowed to grow on designated bacterial strains until day 1 or 4 of adulthood. Worms were then washed three times with M9 buffer and pelleted before addition of 75-80 μL of native lysis buffer (as described in Gidalevitz et al., 2009). Samples were flash frozen in liquid nitrogen prior to use. Nematodes were thawed on ice and mechanically disrupted using a Precellys (Bertin Instruments) programmed for 3x15 s pulses, with 30 s between each pulse. Samples were then centrifuged for 5 minutes (8000 g) and supernatant containing total proteins was transferred to fresh tubes to resolve using NativePAGE 4%–16% Bis-Tris (Invitrogen). Gels were transferred using the iBlot® 7-Minute Blotting System (Thermo Fisher Scientific) and imaged using ChemiDoc (BioRad). ImageLab software (BioRad) and ImageJ were used to analyze band intensity. For denaturing conditions, total cell lysate was prepared in RIPA buffer and samples were boiled at 95°C for 5 minutes prior to loading on NuPAGE 4%–12% Bis Tris gels (Invitrogen). Gels were then transferred and imaged as described above. For both native and denaturing conditions total protein concentrations were assayed using a Pierce BCA protein assay kit (Thermo Scientific).

#### OxyBlot

Age-matched worms were grown to day 1 or day 4 of adulthood at 20°C on 55 mm NGM plates, with or without oleic acid supplementation, seeded with OP50. Animals were then rinsed and collected with M9 buffer. Following this, total cell lysate was prepared as described above and protein oxidation assessed using the OxyBlot protein oxidation detection kit according to the manufacturer’s instruction (Millipore S7150). α-tubulin was used as a loading control, blotting and visualization were performed as described above.

### Quantification and Statistical Analysis

Tests used to determine statistical significance include one-way ANOVA with Dunnett’s multiple comparison test (Oil Red O staining and qRT-PCR analysis); one-way ANOVA with Bonferroni’s post hoc test (GC-MC analysis); two-way ANOVA with Tukey’s multiple comparisons test (chemotaxis assays and western/oxyblot quantification); Mantel-Cox log-rank test (lifespan assays); and Student’s t test (lipase assays and development assays). Statistical information for each experiment can be found in the corresponding figure legend.

## Supplemental Information

Document S1. Figures S1–S4 and Tables S1 and S2

Table S3. Oligonucleotides Used in This Study, Related to STAR Methods

Document S2. Article plus Supplemental Information
