# Supplementary material for: XBP-1 Remodels Lipid Metabolism to Extend Longevity
Source: Cell Rep. 2019 Jul 16;28(3):581–589.e4. doi: 10.1016/j.celrep.2019.06.057 (PMC6656787; doi:10.1016/j.celrep.2019.06.057)
Supplement: Document S1. Figures S1–S4 and Tables S1 and S2 [file mmc1.pdf]

Cell Reports, Volume *28*

Supplemental Information

XBP-1 Remodels Lipid

Metabolism to Extend Longevity

Soudabeh Imanikia, Ming Sheng, Cecilia Castro, Julian L. Griffin, and Rebecca C. Taylor

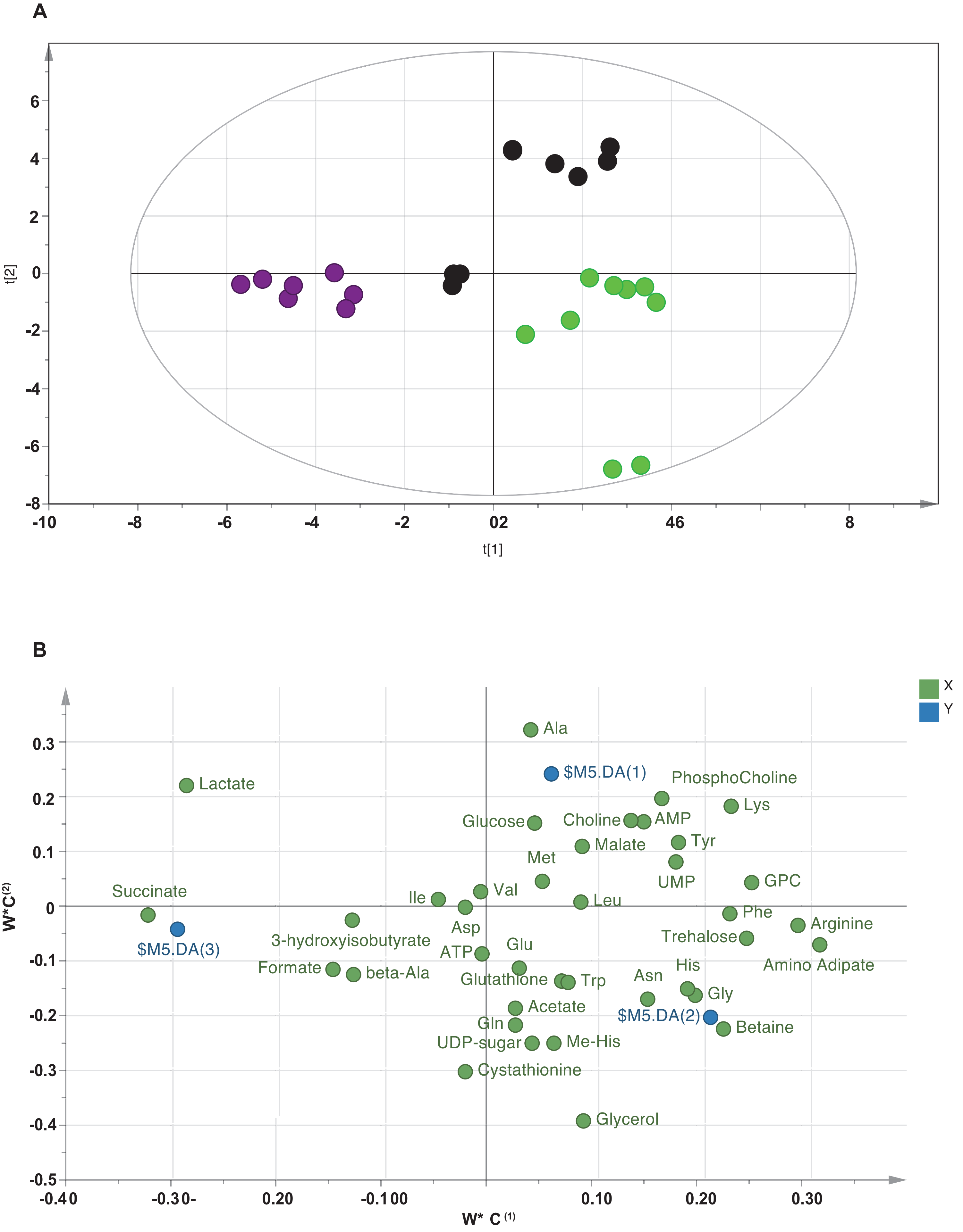
A
6
4
2
0
-2
-4
-6
-8
t[2]
-10
-8
-6
-4
-2
02
46
8
t[1]
B
X
Y
0.3
0.2
0.1
0
-0.1
-0.2
-0.3
-0.4
-0.5
W*C(2)
-0.40
-0.30-
0.20
-0.100
0.10
0.20
0.30
W* C (1)
Ala
$M5.DA(1)
PhosphoCholine
Lactate
Lys
Glucose
Choline
AMP
Malate
Tyr
Met
UMP
GPC
Succinate
Ile
Val
Leu
Phe
Arginine
Asp
Trehalose
$M5.DA(3)
3-hydroxyisobutyrate
ATP
Glu
His
Amino Adipate
Formate
beta-Ala
Glutathione
Trp
Asn
Gly
Acetate
$M5.DA(2)
Gln
Betaine
UDP-sugar
Me-His
Cystathionine
Glycerol

Figure S1. Expression of *xbp-1s* in neurons or the intestine modulates levels of metabolites in *C. elegans*. Related to Figure 1.

(A) Score plot showing clustering pattern according to genotype in metabolic profiles obtained by NMR spectroscopy (ANOVA P-value for the model 1.14E-06). Results from PLS-DA on NMR spectra of N2 (black), *rab-3p::xbp-1s* (green), and *gly-19p::xbp-1s* (purple) animals from 8 independent biological replicates.

(B) Loading plot of individual metabolites altered in *rab-3p::xbp-1s* and *gly-19p::xbp-1s* animals relative to N2. Also see Table S1.

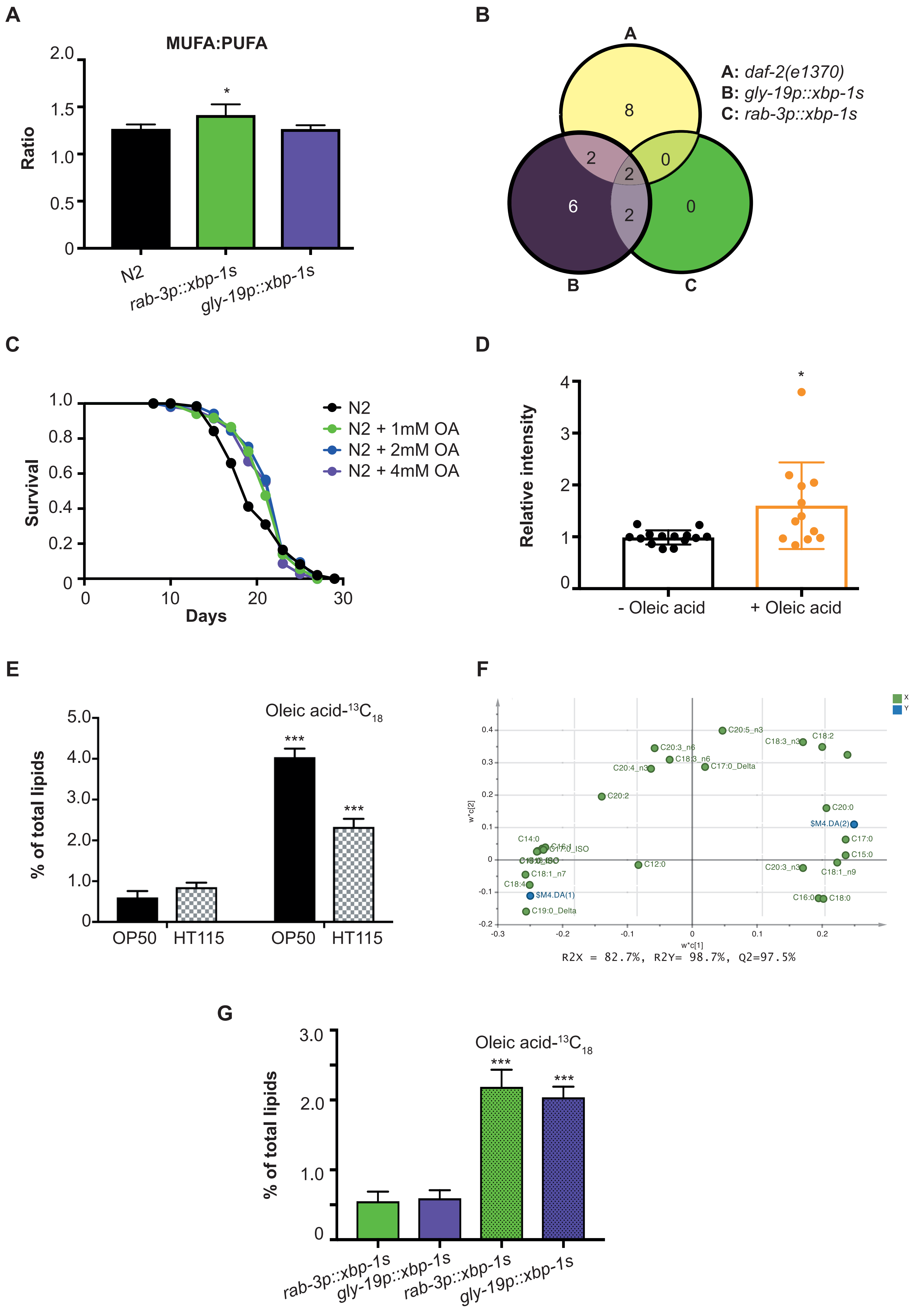
A
MUFA:PUFA
Ratio
2.0
1.5
1.0
0.5
0
*
N2
rab-3p::xbp-1s
gly-19p::xbp-1s
B
A
B
C
8
2
0
2
6
2
0
A: daf-2(e1370)
B: gly-19p::xbp-1s
C: rab-3p::xbp-1s
C
Survival
1.0
0.8
0.6
0.4
0.2
0
0
10
20
30
Days
N2
N2 + 1mM OA
N2 + 2mM OA
N2 + 4mM OA
D
Relative intensity
4
3
2
1
0
*
- Oleic acid
+ Oleic acid
E
Oleic acid-13C18
% of total lipids
5.0
4.0
3.0
2.0
1.0
0
***
***
OP50
HT115
OP50
HT115
F
X
Y
w*c[2]
w*c[1]
C20:5_n3
C18:2
C18:3_n3
C20:3_n6
C18:3_n6
C17:0_Delta
C20:4_n3
C20:2
C20:0
$M4.DA(2)
C17:0
C15:0
C14:0
C16:1
C17:0_ISO
C12:0
C20:3_n3
C18:1_n9
C18:1_n7
C18:4
$M4.DA(1)
C16:0
C18:0
C19:0_Delta
R2X = 82.7%, R2Y= 98.7%, Q2=97.5%
G
Oleic acid-13C18
% of total lipids
3.0
2.0
1.0
0
***
***
rab-3p::xbp-1s
gly-19p::xbp-1s
rab-3p::xbp-1s
gly-19p::xbp-1s

Figure S2. *Xbp-1s*-expressing animals have higher MUFA and OA content, and supplementation with OA successfully increases OA levels. Related to Figure 2.

(A) Ratio of MUFA:PUFA in N2, *rab-3p::xbp-1s* and *gly-19p::xbp-1s* animals. Ratios were calculated from GC-MS analysis of fatty acid species and represent the mean of 7 independent biological replicates ± SEM.

(B) Venn diagram illustrating increased fatty acids relative to N2 in (A) *daf-2(e1370)*, (B) *gly-19p::xbp-1s*, and (C) *rab-3p::xbp-1s*. Also see Table S1.

(C) Lifespan analysis of N2 animals supplemented with 0, 1, 2 or 4mM OA. N2 without OA (black), median lifespan 19 days; N2 with 1mM OA (green), median lifespan 21 days; N2 with 2mM OA (blue), median lifespan 23 days; N2 with 4mM OA (green), median lifespan 23 days. Graphs plotted as Kaplan-Meier survival curves.

(D) Quantification of Oil Red O (ORO) staining of total lipid droplets in N2 animals supplemented with oleic acid. Images were taken of the anterior section of fixed worms acquired at x20 magnification at day 1 of adulthood. Quantification of ORO intensity from two independent experiments was conducted using ImageJ. Plots represent mean relative intensity ± SD. n = 12-14 worms, significance was assessed relative to untreated N2 animals using an unpaired t test, *$p<0.05$.

(E) OA levels in N2 animals grown on *E. coli* OP50 or HT115 bacteria, with and without supplementation with 2mM $^{13}C_{18}$-labelled OA. Levels of labelled OA were measured by GC-MS and plotted as % of total lipids. Data represent the mean ± SD of 8 independent biological replicates. Statistical significance

was calculated using one-way ANOVA with Bonferroni's post hoc test, ***p<0.001.

(F) Loading plot of PLS-DA data showing individual fatty acids in N2 animals supplemented with 2mM OA, compared with animals without supplementation. Nematodes were grown to day 1 of adulthood on *E. coli* OP50. Increased lipid species, including OA (18:1n9), are on the right-hand sector of the graph.

(G) OA levels in *rab-3p::xbp-1s* and *gly-19p::xbp-1s* animals grown on *E. coli* OP50, with and without supplementation with 2mM $^{13}C_{18}$-labelled OA. Levels of labelled OA were measured by GC-MS and plotted as % of total lipids. Data represent the mean ± SD of 8 independent biological replicates. Statistical significance was calculated using one-way ANOVA with Bonferroni's post hoc test, ***p<0.001.

A

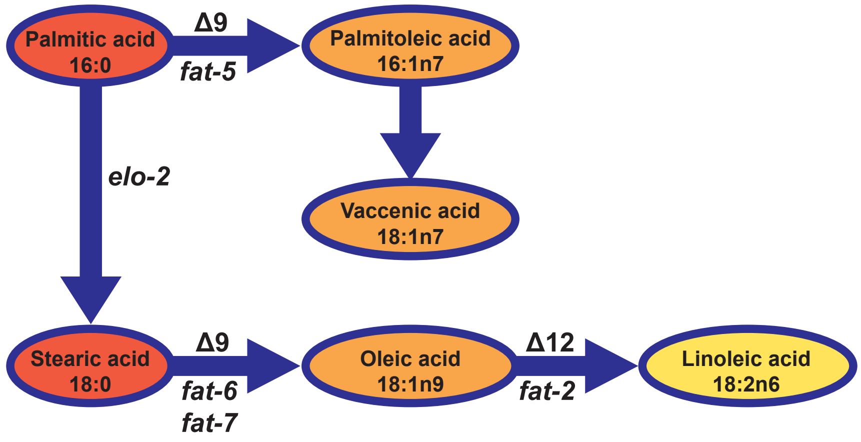

Palmitic acid
16:0
Δ9
fat-5
Palmitoleic acid
16:1n7
elo-2
Vaccenic acid
18:1n7
Stearic acid
18:0
Δ9
fat-6
fat-7
Oleic acid
18:1n9
Δ12
fat-2
Linoleic acid
18:2n6

B

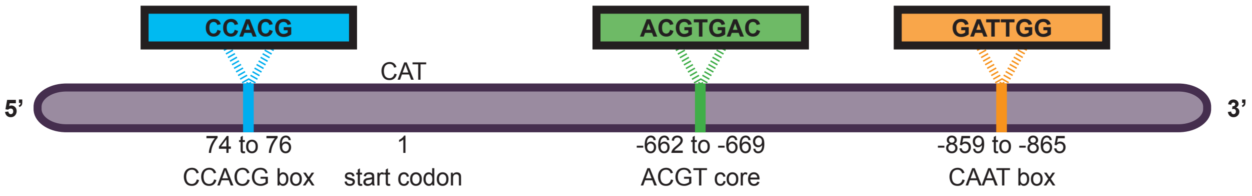

CCACG
ACGTGAC
GATTGG
CAT
5'
3'
74 to 76
CCACG box
1
start codon
-662 to -669
ACGT core
-859 to -865
CAAT box

C

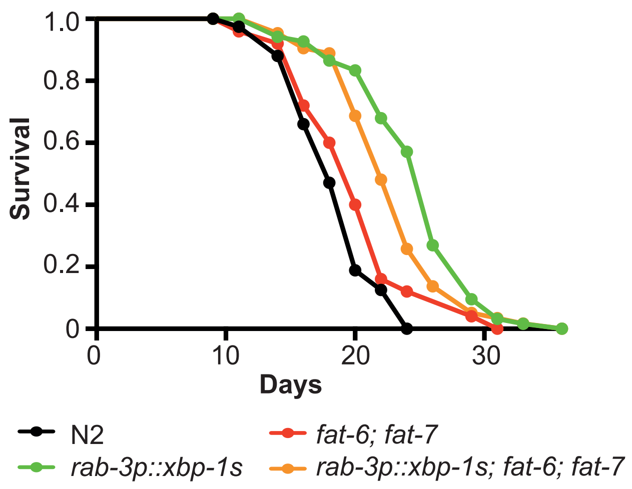

Survival
1.0
0.8
0.6
0.4
0.2
0
0
10
20
30
Days
N2
fat-6; fat-7
rab-3p::xbp-1s
rab-3p::xbp-1s; fat-6; fat-7

Figure S3. The Δ9 desaturase *fat-6* may play a role in *xbp-1s*-induced lifespan extension. Related to Figure 2.

(A) Diagram of oleic acid production in *C. elegans*. Palmitic acid undergoes elongation catalyzed by *elo-2* to produce stearic acid, which is desaturated via the Δ9 desaturases *fat-6* and *fat-7* to produce OA. OA can then be further desaturated by *fat-2* to become linoleic acid. SFAs shown in red, MUFAs in orange, and PUFAs in yellow.

(B) Schematic representation of *xbp-1s* binding motifs in the promoter of *fat-6*. A CCACG box, ACGT core and CAAT box are annotated relative to the *fat-6* start site.

(C) Lifespan analysis of N2, *rab-3p::xbp1s*, *fat-6; fat-7*, and *rab-3p::xbp1s*; *fat-6; fat-7* animals. N2 (black), median lifespan 18 days; *rab-3p::xbp-1s* (green), median lifespan 26 days; *fat-6; fat-7* (red), median lifespan 20 days; *rab-3p::xbp-1s; fat-6; fat-7* (orange), median lifespan 22 days. Graphs plotted as Kaplan-Meier survival curves.

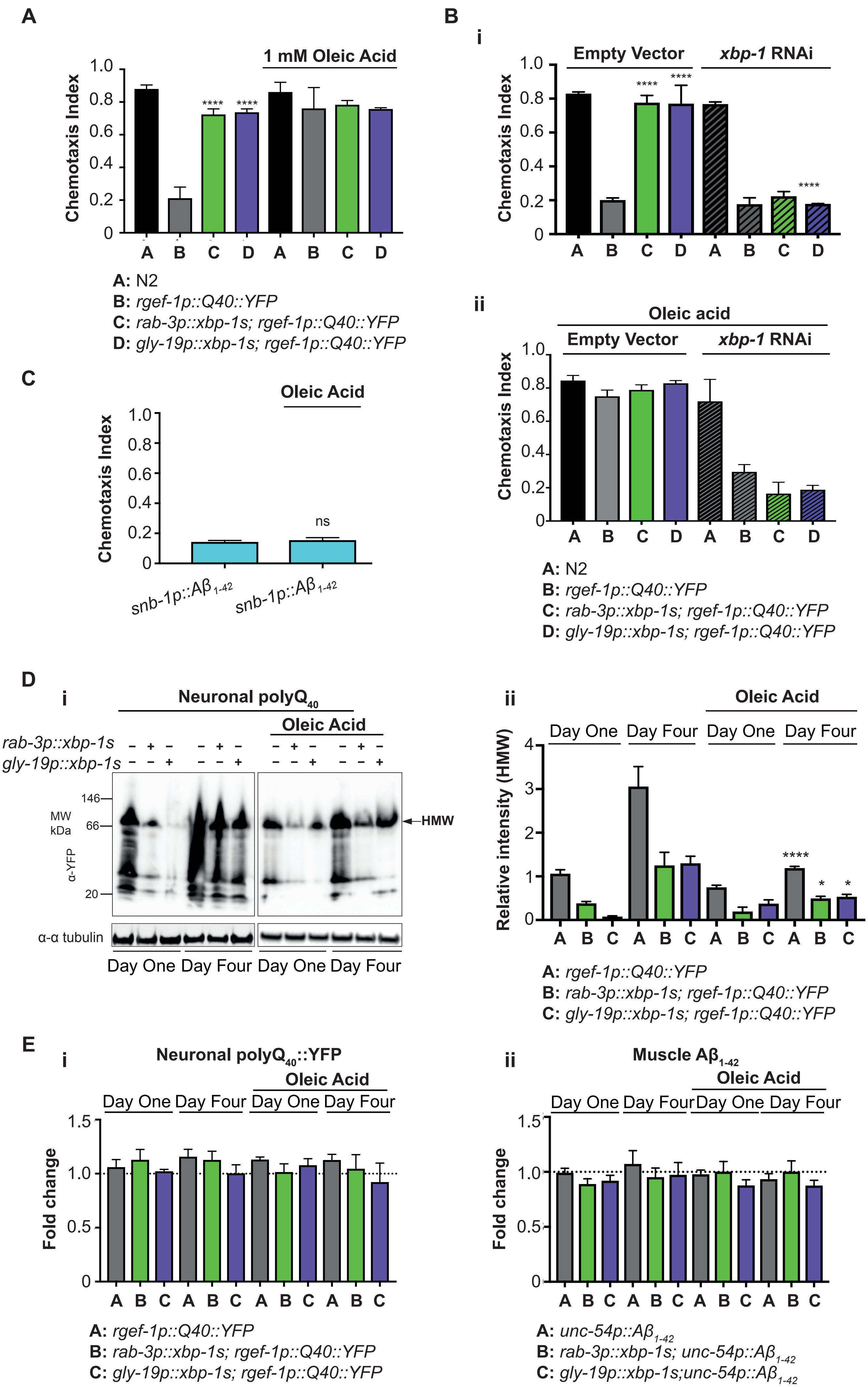
A
1 mM Oleic Acid
Chemotaxis Index
1.0
0.8
0.6
0.4
0.2
0
****
****
A B C D A B C D
A: N2
B: rgef-1p::Q40::YFP
C: rab-3p::xbp-1s; rgef-1p::Q40::YFP
D: gly-19p::xbp-1s; rgef-1p::Q40::YFP
B
i
Empty Vector
xbp-1 RNAi
Chemotaxis Index
****
****
****
A B C D A B C D
ii
Oleic acid
Empty Vector
xbp-1 RNAi
Chemotaxis Index
A B C D A B C D
A: N2
B: rgef-1p::Q40::YFP
C: rab-3p::xbp-1s; rgef-1p::Q40::YFP
D: gly-19p::xbp-1s; rgef-1p::Q40::YFP
C
Oleic Acid
Chemotaxis Index
ns
snb-1p::Aβ1-42
snb-1p::Aβ1-42
D
i
Neuronal polyQ40
Oleic Acid
rab-3p::xbp-1s
gly-19p::xbp-1s
MW kDa
146
66
20
α-YFP
HMW
α-α tubulin
Day One Day Four Day One Day Four
ii
Oleic Acid
Day One Day Four Day One Day Four
Relative intensity (HMW)
4
3
2
1
0
****
*
*
A B C A B C A B C A B C
A: rgef-1p::Q40::YFP
B: rab-3p::xbp-1s; rgef-1p::Q40::YFP
C: gly-19p::xbp-1s; rgef-1p::Q40::YFP
E
i
Neuronal polyQ40::YFP
Oleic Acid
Day One Day Four Day One Day Four
Fold change
1.5
1.0
0.5
0
A B C A B C A B C A B C
A: rgef-1p::Q40::YFP
B: rab-3p::xbp-1s; rgef-1p::Q40::YFP
C: gly-19p::xbp-1s; rgef-1p::Q40::YFP
ii
Muscle Aβ1-42
Oleic Acid
Day One Day Four Day One Day Four
Fold change
A B C A B C A B C A B C
A: unc-54p::Aβ1-42
B: rab-3p::xbp-1s; unc-54p::Aβ1-42
C: gly-19p::xbp-1s;unc-54p::Aβ1-42

Figure S4. Oleic acid protects against proteotoxicity without altering transcript levels of proteotoxic species. Related to Figures 3 and 4.

(A) Chemotaxis ability of worms expressing neuronal polyQ$_{40}$ with and without 1mM OA supplementation. Bar graphs represent mean chemotaxis index ± SD. n = 65-120 animals per assay and each assay was replicated 3 times. Significance was calculated between B and C/D using ordinary two-way ANOVA with Tukey's multiple comparisons test, ****p<0.0001.

(B) (i) Chemotaxis ability of worms expressing neuronal polyQ$_{40}$ in combination with neuronal and intestinal *xbp-1s* and *xbp-1* RNAi, in the absence of OA. Animals were grown on control (empty vector) or *xbp-1* RNAi. Bar graphs represent mean chemotaxis index ± SD. n = 65-120 animals per assay and each assay was replicated 3 times. Significance was calculated between B and C/D using an ordinary two-way ANOVA with Tukey's multiple comparisons test, ****p<0.0001.

(ii) Chemotaxis ability of worms expressing neuronal polyQ$_{40}$ in combination with neuronal and intestinal *xbp-1s* and *xbp-1* RNAi, in the presence of 2mM OA. Animals were grown on control (empty vector) or *xbp-1* RNAi, supplemented with 2mM OA. Bar graphs represent mean chemotaxis index ± SD. n = 65-120 animals per assay and each assay was replicated 3 times. Significance was calculated between B and C/D using an ordinary two-way ANOVA with Tukey's multiple comparisons test, ****p<0.0001.

(C) Chemotaxis ability of worms expressing neuronal Aβ_{1-42} with and without 2mM OA supplementation. Bar graphs represent mean chemotaxis index ± SD. n = 65-120 animals per assay and each assay was replicated 3 times.

Significance was calculated using an ordinary two-way ANOVA with Tukey's multiple comparisons test, ns=not significant.

(D) (i) Western blot analysis of neuronal polyQ$_{40}$::YFP, expressed with and without tissue specific *xbp-1s*, at day 1 and day 4 of adulthood, in the presence and absence of 2mM OA. Lysates containing total proteins were resolved under native conditions and blotted with an anti-YFP/GFP antibody. HMW indicates the higher molecular weight, polyQ-reactive species. Lower molecular weight bands are likely to represent YFP cleaved from polyQ. Tubulin levels were probed with α-α tubulin as a loading control. Data represent two independent experiments.

(ii) Higher molecular weight (HMW) YFP-positive species were quantified from Western blots shown in (i) using ImageJ software. Bar graphs represent mean band intensity relative to day 1 *rgef-1p::Q40::YFP* ± standard deviation (SD). Statistical significance between OA-supplemented versus non-supplemented lanes was assessed using two-way ANOVA with Tukey's multiple comparisons, $^*p<0.05$, $^{**}p<0.01$.

(E) (i) qPCR of YFP transcript levels in animals expressing neuronal polyQ$_{40}$::YFP with and without 2 mM OA supplementation. YFP expression was measured at day 1 and day 4 of adulthood and normalized to unsupplemented day 1 polyQ. Significance was measured by one-way ANOVA with Dunnett's multiple comparisons test.

(D) qPCR of Aβ_{1-42} transcript levels in animals expressing muscle Aβ_{1-42} with and without 2 mM OA supplementation. Aβ_{1-42} expression was measured at day 1 and day 4 of adulthood and normalized to unsupplemented day 1 Aβ_{1-}

[42]. Significance was measured by one-way ANOVA with Dunnett's multiple comparisons test.

Table S1. Metabolic changes in *rab-3p::xbp-1s* and *gly-19p::xbp-1s* animals compared to N2. Related to Figure 1.

Content of fatty acids (% of total lipids) as measured by GC-MS, and levels of other metabolites as measured by NMR. Significance was calculated by students t-test based on 7-8 independent biological replicates. P numbers in red indicate significant difference relative to N2 ($p<0.05$).

GC-MS					
**Fatty Acid**	**N2 (%)**	***rab-3p::xbp-1s* (%)**	***rab-3p::xbp-1s* (P)**	***gly-19p::xbp-1s* (%)**	***gly-19p::xbp-1s* (P)**
C12:0	0.037638125	0.046858922	0.276191445	0.049874632	0.766672155
iso-C13:0	0.069233308	0.061272411	0.202374118	0.03610203	0.202374118
iso-C14:0	0.055922671	0.062674672	0.045658776	0.069696447	0.161431178
C14:0	0.456966786	0.44690672	0.001271845	0.659629622	1.36789E-08
iso-C15:0	2.903133726	2.913185434	0.096044766	3.129290683	0.504949798
ante-C15:0	1.876237705	2.182870005	0.922263801	0.968955147	9.24518E-06
C15:0	0.05839206	0.038281908	0.25839068	0.044060149	0.262393056
iso-C16:0	1.113005128	1.100065998	0.103039632	0.39289377	5.93293E-07
C16:0	3.642157426	3.636536533	0.255133332	7.316688704	1.57692E-06
C16:1	1.379773152	1.129854757	0.77652476	1.10902079	0.224580943
iso-C17:0	4.942977679	4.72343748	0.249957091	3.938632668	1.12147E-05
ante-C17:0	2.1627657	2.281910758	0.046274702	0.587095443	1.13881E-08
C17:0	0.175129884	0.180060078	0.101038088	0.232162779	0.010577341
delta-C17:0	11.00595127	11.82852658	0.10719598	10.77852968	0.857068107
C18:0	6.021720233	6.053582553	0.485454425	9.515798457	2.42849E-08
C18:1n9	2.091876787	2.396176071	0.0262	2.739179844	0.00085799
C18:1n7	21.66477177	21.30188965	0.540674927	17.65895357	7.91517E-07
C18:2	6.878844605	7.045406611	0.242050229	7.813655733	0.000116917
delta-C19:0	5.908140253	6.618385551	0.078752592	7.725027412	0.002364508
C18:3n3	0.417336489	0.501618839	0.148073074	0.584215629	3.77989E-05
C20:0	0.293329949	0.372224452	0.013235386	0.561160607	9.98428E-12
C18:4	0.084832823	0.134347219	0.004834874	0.241255089	3.50182E-10
C20:2n6	1.705329629	1.770562627	0.49905635	1.00348906	6.87085E-05
C20:3n6	2.710177904	2.4312682	0.113875688	2.654929741	0.000132048
C20:4n6	1.062258484	0.944843771	0.229003759	1.164554506	1.68142E-05
C20:3n3	0.281116993	0.3032934	0.977921443	0.219770886	0.000142186
C22:0	0.1717916	0.210536154	0.970371469	0.287594445	0.328412194
C20:4n3	5.041856388	4.520798296	0.000574296	4.155949497	0.000550553
C20:5n3	15.78733146	14.76262435	0.000840718	14.36183297	0.033976502
**NMR**					
**Metabolite**	**N2 (loading value)**	***rab-3p::xbp-1s* (loading value)**	***rab-3p::xbp-1s* (P)**	***gly-19p::xbp-1s* (loading value)**	***gly-19p::xbp-1s* (P)**
Acetate	2.66612E-05	6.0923E-05	0.011	0.000279359	0.0895
Ala	3.71315E-05	0.000110882	0.071	0.000507694	0.0571
Amino Adipate	3.55234E-05	8.39983E-05	0.0085	0.000415428	0.0769
AMP	0.000132062	0.000251237	0.0485	0.001318336	0.0568
Arginine	3.6126E-05	7.98851E-05	0.1263	0.000426406	0.0505
Asn	0.000335146	0.000540685	0.0238	0.003784171	0.0513
Asp	0.000106686	0.000228942	0.2445	0.000778061	0.0597
beta-Ala	7.57842E-05	0.000118469	0.0145	0.000926281	0.0554
Betaine	3.40317E-05	6.88834E-05	0.1483	0.000171534	0.0582
Cystathionine	0.000150441	0.000236104	0.0009	0.00278822	0.0894
Formate	6.59597E-05	0.000179191	0.3396	0.000257923	0.113
Gln	6.11792E-05	0.000109712	0.0233	0.00085101	0.0512
Glu	2.95155E-05	5.54915E-05	0.0769	0.000376749	0.0525
Glucose	7.05752E-06	1.49685E-05	0.0279	0.000158892	0.0279
Glutathione	1.68392E-05	4.94249E-05	0.029	0.000223008	0.048
Gly	1.51044E-05	3.01106E-05	0.1169	0.000201218	0.0531
Glycerol	2.12337E-05	2.93477E-05	0.0023	0.000464913	0.9183
GPC	2.41995E-05	4.73906E-05	0.2843	0.000437628	0.082
His	1.97093E-05	3.44798E-05	0.093	0.000272015	0.0584
Ile	1.94402E-05	2.31847E-05	0.0127	0.000308325	0.065

Lactate	1.83265E-05	1.29755E-05	0.0355	0.000193492	0.0605
Leu	8.29979E-05	0.000120555	0.0018	0.001354631	0.0493
Lys	2.09462E-05	3.07703E-05	0.0675	0.000245048	0.0578
Me-His	4.65184E-05	6.37157E-05	0.0118	0.000372417	0.0536
Met	6.0956E-05	0.000103229	0.001	0.000296547	0.0699
Phe	2.68098E-05	4.19086E-05	0.0127	0.000419872	0.0593
PhosphoCholine	9.48826E-05	0.000155596	0.8025	0.001865628	0.082
Succinate	1.83553E-05	3.14121E-05	0.0552	0.000226714	0.0034
Thr	1.16742E-05	2.16723E-05	0.0078	0.000318934	0.0571
Trehalose	1.30484E-05	2.63587E-05	0.1189	0.000166687	0.0471
Trp	1.97375E-05	3.32716E-05	0.0138	0.00017208	0.0386
Tyr	2.75897E-05	5.49874E-05	0.0171	0.000353804	0.0498
UDP-sugar	1.09234E-05	2.18598E-05	0.0461	0.000113759	0.0521
Val	4.77777E-06	6.77345E-06	0.0085	2.84092E-05	0.0622

Table S2. Survival information for lifespan experiments. Related to Figures 1 and 2.

Strain	Condition	Median lifespan (days)	Control	Condition	Median lifespan (days)	P value (Mantel-Cox)
N2	2mM OA	23	N2	-	21	<0.0001
N2	2mM OA	26	N2	-	21	<0.0001
N2	2mM OA	20	N2	-	18	<0.0001
rab-3p::xbp-1s	2mM OA	25	*rab-3p::xbp-1s*	-	25	0.2947
rab-3p::xbp-1s	2mM OA	29	*rab-3p::xbp-1s*	-	33	<0.0001
rab-3p::xbp-1s	2mM OA	26	*rab-3p::xbp-1s*	-	28	<0.0001
gly-19p::xbp-1s	2mM OA	23	*gly-19p::xbp-1s*		23	0.4600
gly-19p::xbp-1s	2mM OA	23	*gly-19p::xbp-1s*		23	0.0246
gly-19p::xbp-1s	2mM OA	21	*gly-19p::xbp-1s*		21	0.8658
xbp-1(zc12)	2mM OA	22	*xbp-1(zc12)*	-	18	<0.0001
xbp-1(zc12)	2mM OA	18	*xbp-1(zc12)*	-	16	0.0002
xbp-1(zc12)	2mM OA	18	*xbp-1(zc12)*	-	16	<0.0001
rab-3p::xbp-1s; fat-6; fat-7	-	22	*rab-3p::xbp-1s*	-	26	0.0134
rab-3p::xbp-1s; fat-6; fat-7	-	25	*rab-3p::xbp-1s*	-	27	0.0417
rab-3p::xbp-1s; fat-6; fat-7	-	24	*rab-3p::xbp-1s*	-	29	0.0034
rab-3p::xbp-1s; fat-6; fat-7	-	23	*rab-3p::xbp-1s*	-	29	<0.0001
rab-3p::xbp-1s; fat-6; fat-7	-	25	*rab-3p::xbp-1s*	-	27	0.0102
rab-3p::xbp-1s; fat-6; fat-7	-	25	*rab-3p::xbp-1s*	-	27	0.0464
rab-3p::xbp-1s; fat-6; fat-7	2mM OA	28	*rab-3p::xbp-1s*	-	29	0.8152
rab-3p::xbp-1s; fat-6; fat-7	2mM OA	27	*rab-3p::xbp-1s*	-	27	0.1638
rab-3p::xbp-1s; fat-6; fat-7	2mM OA	27	*rab-3p::xbp-1s*	-	27	0.2021
